# What to Observe at the Bed-Side and after Death in Medical Cases

**Published:** 1853-06

**Authors:** 


*What to observe at the bed-side and after death in Medical cases.* Published under the authority of the London Medical Society of Observation. Philadelphia: Blanchard & Lea. 1853. 12mo.

This little work contains a list and systematic arrangement of all the points of observation in studying the phenomena of disease at the bed-side. By enabling the observer to take a comprehensive view of the subjects to which his attention should be directed in prosecuting clinical studies, the work will prove useful, serving both as a guide, and remembrancer. But to carry out the plan, with all its details, in recording cases of disease, would be almost impracticable. Neither patient nor physician would have patience enough to collect many histories embracing information respecting all the particulars enumerated in the work. For the objects just stated, it appears to us to be a valuable book for reference. It has been prepared by Dr. Walshe, of London, himself an excellent observer.

